# Crosstalk between hepatic tumor cells and macrophages via Wnt/β-catenin signaling promotes M2-like macrophage polarization and reinforces tumor malignant behaviors

**DOI:** 10.1038/s41419-018-0818-0

**Published:** 2018-07-18

**Authors:** Yang Yang, Yu-Chen Ye, Yan Chen, Jun-Long Zhao, Chun-Chen Gao, Hua Han, Wen-Chao Liu, Hong-Yan Qin

**Affiliations:** 1 State Key Laboratory of Cancer Biology, Department of Medical Genetics and Developmental Biology, Fourth Military Medical University, 710032 Xi’an, China; 2 Department of Clinical Oncology, Xijing Hospital, Fourth Military Medical University, 710032 Xi’an, China

## Abstract

Tumor-associated macrophages (TAMs) are a major component of tumor microenvironment (TME) and play pivotal roles in the progression of hepatocellular carcinoma (HCC). Wnt signaling is evolutionarily conserved and participates in liver tumorigenesis. Several studies have shown that macrophage-derived Wnt ligands can activate Wnt signaling in tumor cells. However, whether Wnt ligands secreted by tumor cells can trigger Wnt signaling in macrophages is still elusive. In this study, we first verified that canonical Wnt/β-catenin signaling was activated during monocyte-to-macrophage differentiation and in M2-polarized macrophages. Knockdown of β-catenin in M2 macrophages exhibited stronger antitumor characteristics when cocultured with Hepa1-6 HCC cells in a series of experiments. Activation of Wnt signaling promoted M2 macrophage polarization through c-Myc. Moreover, co-culturing naïve macrophages with Hepa1-6 HCC cells in which Wnt ligands secretion was blocked by knockdown of Wntless inhibited M2 polarization in vitro. Consistently, the growth of HCC tumor orthotopically inoculated with Wntless-silenced Hepa1-6 cells was impeded, and the phenotype of M2-like TAMs was abrogated due to attenuated Wnt/β-catenin signaling in TAMs, leading to subverted immunosuppressive TME. Finally, we confirmed the correlation between M2 macrophage polarization and nuclear β-catenin accumulation in CD68^+^ macrophages in human HCC biopsies. Taken together, our study indicates that tumor cells-derived Wnt ligands stimulate M2-like polarization of TAMs via canonical Wnt/β-catenin signaling, which results in tumor growth, migration, metastasis, and immunosuppression in HCC. To block Wnts secretion from tumor cells and/or Wnt/β-catenin signal activation in TAMs may be potential strategy for HCC therapy in future.

## Introduction

Hepatocellular carcinoma (HCC) is one of the most common and aggressive inflammation-related human cancers in the world^1^. Recently, inflammation has been highlighted as the seventh hallmark of cancer, which establishes the relationship between tumor cells and the tumor microenvironment (TME)^2^. As a major component of TME, tumor-associated macrophages (TAMs) play a pivotal role in the progression of inflammation-related cancers, including HCC^3,4^. Many studies have indicated that TAMs promote tumor initiation, angiogenesis, metastasis, and suppression of adaptive immunity through the production of a large amount of cytokines, chemokines, growth factors and matrix metalloproteases in TME^5,6^. Indeed, infiltrated TAMs are associated with poor prognosis of HCC patients^7,8^. These studies suggest that TAMs can be a potential target for HCC therapy.

TAMs possess high heterogeneity, which can be ascribed to their origin and activation status and function^9^. Under inflammatory stimulation, monocytes are recruited to injured tissue and differentiate into macrophages with differently polarized activation states. Activation with interferon-gamma (IFN-γ), or IFN-γ combined with lipopolysaccharide (LPS) polarizes macrophages into classically activated macrophages, namely M1 macrophages, which develop the proinflammatory Th1 immune response and exert tumoricidal activity by the expression of high levels of proinflammatory cytokines, such as interleukin (IL)-6, tumor necrosis factor (TNF)-α, and high production of reactive nitrogen and oxygen intermediates (RNS and ROS), respectively. In contrast to M1 macrophage polarization, IL-4/IL-13, IL-10 or TGF-β induces macrophages to polarize into alternatively activated macrophages, referred to as M2 macrophages, which are associated with the anti-inflammatory Th2 immune response and possess protumor activity by high expression of mannose receptor (MR), arginase1 (Arg1) and Ym1^10^. In most tumors, the characteristics of TAMs are similar to M2 macrophages in several aspects, and therefore, TAMs are also called M2-like macrophages^11^. Currently, the molecular mechanisms of macrophage polarization have been explained at different levels, including signaling pathways, transcription factors, and epigenetic regulation^12^. However, the detailed mechanisms underlying the crosstalk between tumor cells and macrophage polarization in TME remains largely unknown.

Growing evidence shows that crosstalk between tumor cells and macrophages is involved in tumor progression^6^. Many kinds of soluble factors, such as Wnts, are important for regulating cell−cell interaction^13^. Wnt ligands are secreted proteins that not only participate in cellular proliferation, migration and tissue patterning during embryonic development, but also are involved in many diseases, especially tumorigenesis^14–16^. Generally, Wnt ligands can be secreted into the extracellular milieu controlled by Wntless, and then bind to the Frizzled receptors on the signal-competent cells to induce the canonical Wnt/β-catenin signaling or noncanonical Wnt/Ca2^+^ signaling in paracrine/autocrine manners^17^. Several studies have shown an autocrine mechanism for constitutive Wnt pathway activation in human cancer cells including breast cancer, ovarian cancer, and non-small cell lung carcinoma^18,19^. In contrast, Binder’s and Pollard’s groups found that a paracrine Wnt signaling loop exists between breast tumor cells and TAMs using in vitro and in vivo assays, as Wnt ligands can also be expressed by macrophages^20,21^. Moreover, Cosin-Roger et al. reported that Wnt ligands from M2 macrophages activate Wnt signaling in intestinal epithelial cells^22^. It is known that Wnt/β-catenin signaling plays important roles in liver development, regeneration, and cancer, and that Wnt ligands and receptors can be expressed by various hepatic cell types, such as hepatocytes and Kupffer cells (KCs) that are one kind of tissue resident macrophages^23^. Recently, Boulter et al. have found that macrophage-derived Wnt3a opposes Notch signaling to promote hepatic progenitor cell specification to hepatocytes in chronic liver diseases^24^. Therefore, we wondered whether hepatic tumor cells-derived Wnt ligands can trigger Wnt/β-catenin signaling in macrophages through a paracrine manner.

In this study, we report that the Wnt/β-catenin signaling was activated during monocyte-to-macrophage differentiation and in M2 macrophages. Activated Wnt/β-catenin signaling promoted M2 macrophage polarization, which elicited M2-like phenotype of TAMs in coculture with Hepa1-6 HCC cells. Further investigation showed that Wnt/β-catenin signaling regulated M2 macrophage polarization through c-Myc. Blocking Wnt ligands secretion from Hepa1-6 cells by Wntless knockdown inhibits M2 macrophage polarization in vitro and reduces tumor growth in vivo by reprogramming the tumor immune microenvironment. Finally, the correlation between M2 macrophage polarization and nuclear β-catenin accumulation in TAMs was verified in HCC patient biopsies. Taken together, our study for the first time showed that hepatic tumor cells promoted M2 macrophage polarization through Wnt/β-catenin signaling in a paracrine manner. Blocking Wnt secretion from hepatic tumor cells and/or Wnt/β-catenin signal activation in TAMs may be a promising strategy for liver cancer therapy.

## Results

### Wnt/β-catenin signaling was activated during monocytes differentiation into macrophages and was highly expressed in M2-polarized macrophages

Several studies have shown that Wnt/β-catenin signaling regulates differentiation of human monocytes into macrophages^25–27^. Therefore, we assessed the possibility whether Wnt/β-catenin signaling is involved in the differentiation of monocytes into macrophages in mice. CD11b^+^ monocytes were sorted from mouse bone marrow (BM) by magnetic activated cell sorting, and then were induced into macrophages with M-CSF stimulation for 7 days. The purity of the monocytes and macrophages was approximately 98% by FACS assay (Supplement Fig. 1A and B). Then, the expression level of Wnt signal-associated molecules in monocytes and macrophages was detected using qRT-PCR. The result showed that the expression of β-catenin, c-Myc, and cyclin D1 was significantly increased in mature macrophages (Supplement Fig. 1C), suggesting that Wnt signaling was activated during monocytes differentiation into macrophages.

**Fig. 1:**
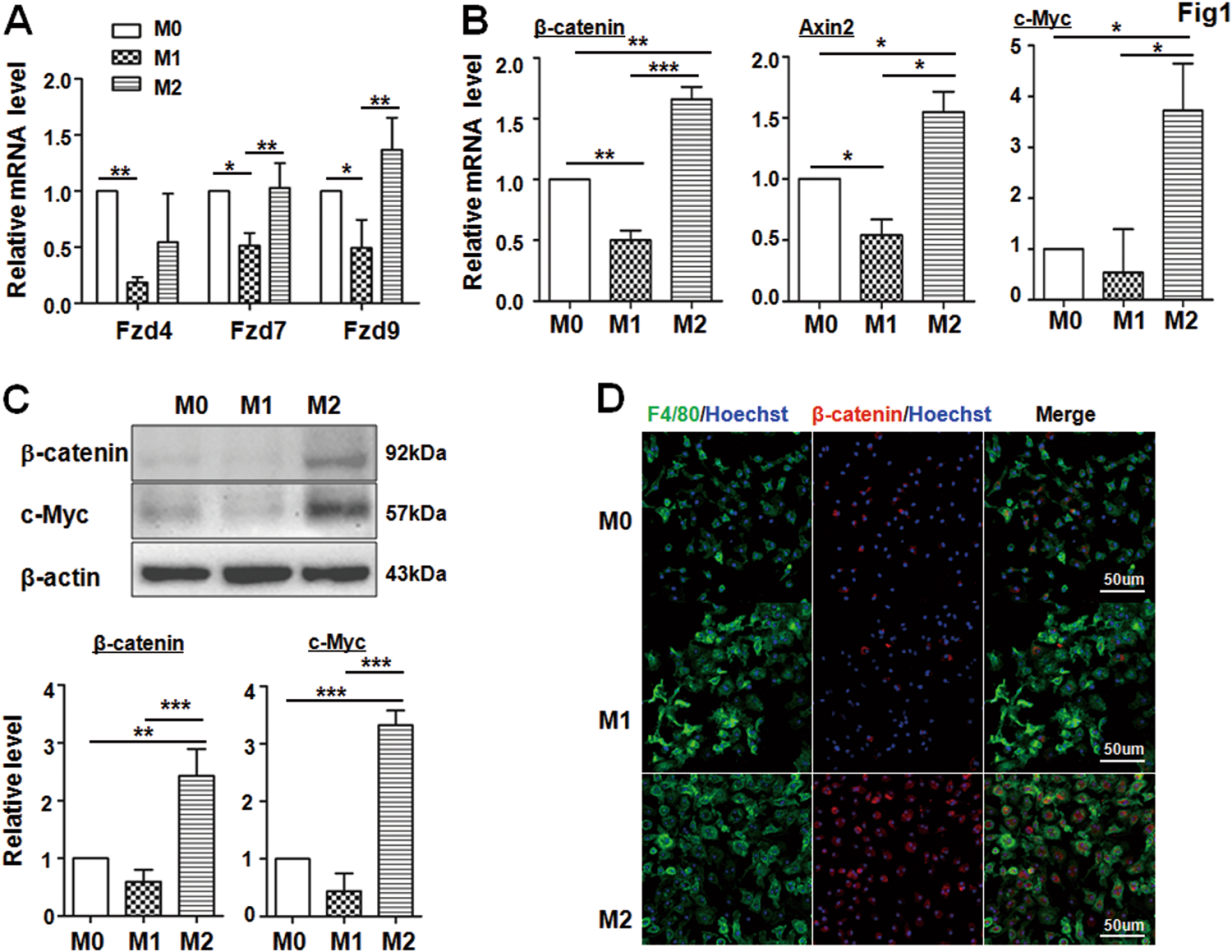
Wnt/β-catenin signaling is expressed in M2 macrophages. **a**, **b** BMDMs (M0) were stimulated with LPS + IFN-γ (M1) or IL-4 (M2). The expression of receptors (**a**) and the downstream genes of Wnt signaling (**b**) were determined by qRT-PCR, using β-actin as an internal control. A quantitative comparison among M0, M1, and M2 macrophages was presented (*n* = 5). **c** The protein level of downstream genes of Wnt signaling including β-catenin and c-Myc was detected by western blotting, and then quantified among M0, M1, and M2 macrophages (*n* = 3). **d** The expression of β-catenin in different polarized BMDMs was detected by immunofluorenscence (IF) staining. Cell nuclei were counterstained with Hoechst (*n* = 3). Bars, mean ± SD; *P < 0.05; **P < 0.01; ***P < 0.001

Next, after the BM-derived macrophages (BMDMs, namely, M0) were polarized to M1 or M2 phenotype under LPS + INF-γ or IL-4 stimulation, respectively, we examined the mRNA expression of Wnt receptors and downstream genes of Wnt signaling in M0, M1 and M2 BMDMs by qRT-PCR. We found that the expression level of Wnt receptors such as Fzd7 and Fzd9 was lower in M1 macrophages but comparable in M2 macrophages, suggesting that Wnt signaling could be involved in macrophage polarization (Fig. 1a). Indeed, the mRNA levels of three downstream genes of the Wnt/β-catenin signaling pathway including β-catenin, Axin2, and c-Myc were significantly increased in M2 macrophages compared with that in M0 or M1 macrophages (Fig. 1b). Consistently, the protein levels of β-catenin and c-Myc were also significantly upregulated in M2 macrophages as determined by western blotting (Fig. 1c). Moreover, immunofluorescence staining showed an increased positive signal of β-catenin in the nuclei of M2 macrophages (Fig. 1d). Taken together, these results suggested that Wnt/β-catenin signaling was involved in monocytes differentiation and was highly activated in M2 macrophages.

### Wnt/β-catenin signaling promoted M2 macrophage polarization irrespective of M1 or M2 inducers

**Fig. 2:**
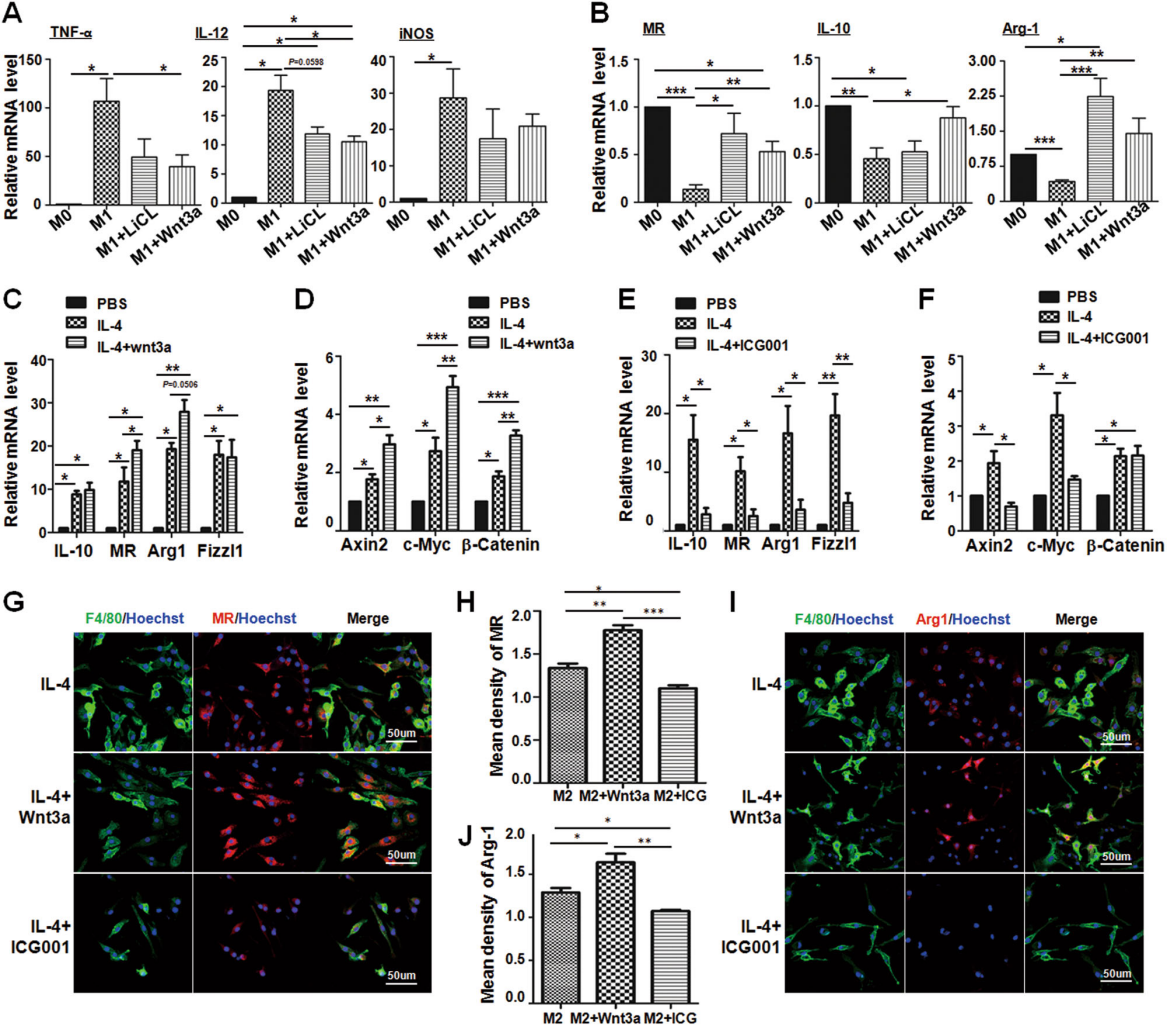
Wnt/β-catenin signaling activation promotes M2 macrophages polarization. **a**, **b** BMDMs (M0) were treated with Wnt3a (100 ng/mL) or LiCl (10 mM) in advance. Cells were then stimulated with LPS + IFN-γ (M1) for 24 h. The mRNA levels of M1 markers such as TNF-α, IL-12, and iNOS (**a**), and M2 markers, including Arg1, MR and IL-10 (**b**), were determined by qRT-PCR, respectively. β-actin was used as an internal control (*n* = 3). **c**, **d** BMDMs were polarized with IL-4 (M2) followed by Wnt3a (100 ng/mL) treatment or no treatment for 24 h. The mRNA level of M2 markers (**c**) and the downstream genes of Wnt signaling (**d**) were detected by qRT-PCR, and then were quantitatively compared (*n* = 3). **e**, **f** Wnt signaling inhibitor ICG001 (10 μM) was added into BMDMs, and then BMDMs were polarized with IL-4 for 24 h. The mRNA level of M2 markers (**e**) and the downstream genes of Wnt signaling (**f**) were examined by qRT-PCR, and then compared quantitatively (*n* = 3). **g**−**j** BMDMs were cultured on coverslips, and treated with Wnt3a or ICG001 followed by IL-4 stimulation for 24 h. Then cells were subjected to IF staining using anti-F4/80 and anti-MR (**g**, **h**) or anti-Arg1 (**i**, **j**) antibodies. Nuclei were counterstained with Hoechst (*n* = 3). Mean florescence intensity was determined and compared. Bars, mean ± SD; *P < 0.05; **P < 0.01; ***P < 0.001

To explore the role of activated Wnt/β-catenin signaling in macrophage polarization, Wnt3a, which is the classical agonist of Wnt/β-catenin signaling^28^, or lithium chloride (LiCl)^29^ which is a GSK-3β inhibitor, were added into BMDM culture followed by LPS + INF-γ treatment for 24 h. The result showed that the expression of M1 macrophage surface markers such as TNF-α and IL-12 was significantly reduced, and the functional marker iNOS was also reduced slightly under M1 status with Wnt3a or LiCl treatment (Fig. 2a). Meanwhile, the expression of M2 macrophage-related molecules, such as MR, IL-10 and Arg1 was significantly increased in the same situation (Fig. 2b), suggesting that Wnt signal activation might promote macrophage M2 polarization under inflammatory stimulation. Moreover, the expression of M2-related molecules in macrophages was increased significantly after IL-4 treatment (Fig. 2c, e), and Wnt3a stimulation further upregulated MR and probably also Arg1 as compared with IL-4-treated macrophages (Fig. 2c). Meanwhile, the expression of Wnt signal downstream genes including Axin2, c-Myc, and β-catenin was enhanced remarkably after IL-4 or IL-4 plus Wnt3a stimulation, especially in IL-4 plus Wnt3a stimulation group (Fig. 2d).

We then examined M2 macrophage polarization after blockade of Wnt/β-catenin signaling with ICG001, an antagonist of Wnt/β-catenin signaling^30^. ICG001 treatment dramatically reduced the expression of M2 macrophage-related molecules following IL-4 stimulation (Fig. 2e). Meanwhile, the activated Wnt/β-catenin signaling was successfully inhibited in the ICG001 plus IL-4 group (Fig. 2f). Moreover, the effect of Wnt/β-catenin signaling on M2 macrophage polarization was further examined by immunofluoresence staining with F4/80 plus MR or Arg1 antibodies. As shown in Fig. 2g−j, compared with IL-4 treatment alone, the MR^+^F4/80^+^ or Arg1^+^F4/80^+^ macrophages increased significantly under IL-4 plus Wnt3a treatment, whereas these M2 macrophages decreased markedly with ICG001 treatment. These results demonstrated that Wnt/β-catenin signaling promoted M2 macrophage polarization irrespective of M1 or M2 inducers.

### Wnt/β-catenin activation promoted M2 macrophage polarization through c-Myc

**Fig. 3:**
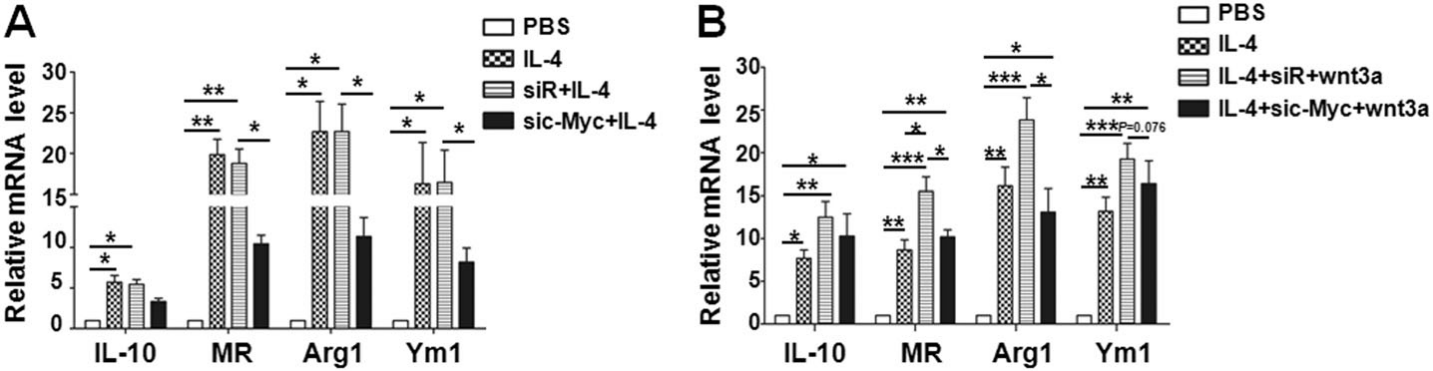
Activated Wnt/β-catenin signaling regulates M2 macrophage polarization through c-Myc. **a** BMDMs were transfected with c-Myc siRNA (sic-Myc) or randomized siRNA control (siR) for 24 h, and then the cells were stimulated with IL-4 for 24 h. The expression of M2 markers was determined by qRT-PCR, and compared quantitatively among each group (*n* = 3). **b** BMDMs were transfected with sic-Myc or siR for 24 h, and then the cells were polarized with IL-4 in the presence of Wnt3a for 24 h. The expression of M2 markers was determined by qRT-PCR, and compared quantitatively among each group (*n* = 3). Bars, mean ± SD; *P < 0.05; **P < 0.01; ***P < 0.001

Recently, Pello et al. have reported that c-Myc is a key player in alternative or M2 macrophage activation^31^. Because c-Myc is a common target gene of Wnt/β-catenin signaling^32^, we wondered whether Wnt/β-catenin signaling promoted M2 macrophages through c-Myc. To test this hypothesis, three siRNAs against c-Myc were synthesized and transfected into BMDMs. The efficiency of knockdown c-Myc in BMDMs was examined by qRT-PCR and western blotting, and two c-Myc siRNA efficiently repressed c-Myc expression (Supplement Fig. 2A and B). Next, c-Myc siRNA (sic-Myc) or randomized control siRNA (siR) was transfected into BMDMs 24 h before IL-4 stimulation. Consistent with the effect of ICG001 (Fig. 2e), the expression of M2 macrophage-associated markers such as MR, Arg1, and Ym1 was reduced significantly in the sic-Myc plus IL-4 group compared with that in the control group (Fig. 3a). Similarly, promotion of M2 macrophages by Wnt3a was abrogated after sic-Myc treatment (Fig. 3b), demonstrating that c-Myc was responsible for M2 macrophage polarization mediated by Wnt/β-catenin signaling.

### Blockade of Wnt/β-catenin signaling in M2 macrophages abrogated TAMs phenotypes in vitro

Growing evidence shows that TAMs are M2-like macrophages^11^. Thus, we sorted TAMs from hepatic tumors in Hepa1-6-bearing mice, as well as KCs from normal mice as a control. The purity of sorted macrophages was approximately 92% assayed by FACS (Supplement Fig. 3A). The expression of M1 and M2 macrophage-related molecules was detected in TAMs and KCs by qRT-PCR. The result showed that TAMs in hepatic tumor indeed expressed higher level of M2-related markers as compared with KCs from normal mice (Supplement Fig. 3B). Moreover, we found that Wnt/β-catenin signaling was also activated in TAMs as compared with KCs from normal mice (Supplement Fig. 3C). We next started to examine whether Wnt/β-catenin signaling could regulate TAM biological behaviors.

**Fig. 4:**
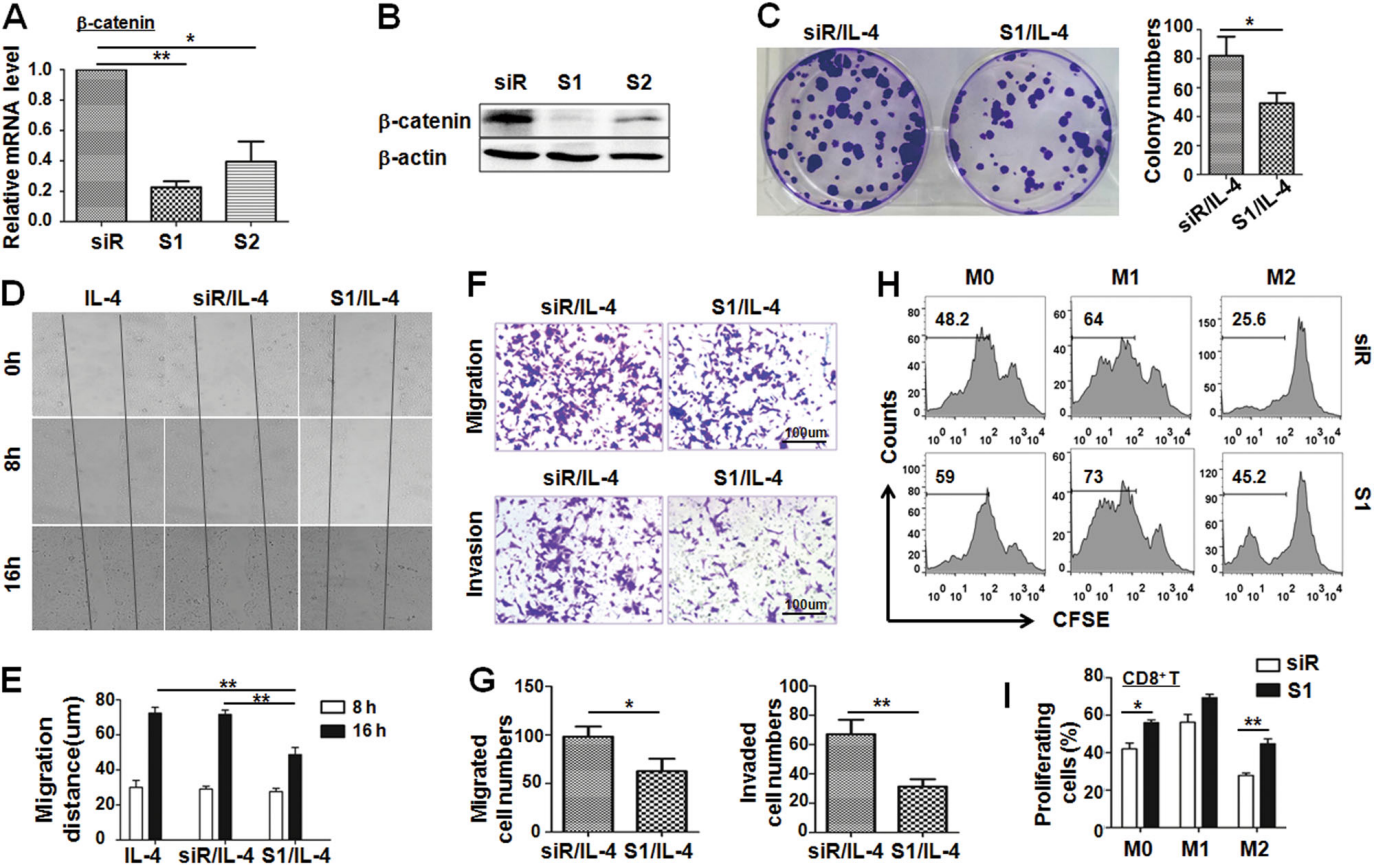
Knockdown Wnt/β-catenin signaling in M2 macrophages abrogates protumor function of macrophages. **a**, **b** BMDMs were transfected with β-catenin siRNA (S1 or S2) or control oligo (siR) for 24 h. The expression level of β-catenin was determined by qRT-PCR (**a**) and western blotting (**b**) (*n* = 3). **c** Knockdown of β-catenin in M2 BMDMs inhibited the ability of colony-forming ability of Hepa1-6 cells in the co-culture system. Representative images were taken at 14 days after incubation. The number of Hepa1-6 colonies was counted and compared (*n* = 3). **d**, **e** Knockdown of β-catenin in M2 BMDMs slowed down the wound-healing ability of Hepa1-6 cells in the co-culture system as determined by the scratch wound assay. The representative images of scratch wound healing were shown in (**d**). The migration distance of Hepa1-6 cells between two scratches was measured and calculated (**e**) (*n* = 3). **f**, **g** Knockdown of β-catenin in M2 BMDMs reduced the ability of migration (**f**, upper panel) and invasion (**f**, lower panel) of Hepa1-6 in the co-culture system as determined by a Transwell assay. The number of migration (**g**, left panel) and invasion (**g**, right panel) of Hepa1-6 cells was counted and compared (*n* = 3). **h**, **i** Knockdown of β-catenin was performed in differentially polarized macrophages that were irradiated with 20 Gy X-rays. Then, the irradiated cells were co-cultured with CFSE-labeled allogenic T cells for 5 days. The proliferation of T cells was determined by FACS (**h**) and was quantitatively compared among different groups (**i**) (*n* = 3). Bars, mean ± SD; *P < 0.05; **P < 0.01

β-catenin is a key player of canonical Wnt signaling. To observe the role of canonical Wnt signaling in the M2 macrophage phenotype, we silenced β-catenin expression in M2 macrophages by siRNAs and found that si-β-catenin (S1) efficiently repressed β-catenin expression in BMDMs by qRT-PCR and western blotting (Fig. 4a, b). Because TAMs play important roles in tumor cell proliferation, invasion, metastasis, and immunosuppression, we first detected the ability of M2 macrophages with β-catenin knockdown to promote tumor cell proliferation by a colony-forming assay. The result showed that the colony number of Hepa1-6 cells cultured with conditional medium (CM) from M2 macrophages with β-catenin knockdown (S1) was significantly less than that of Hepa1-6 cultured with CM from the control (siR) (Fig. 4c). Second, we measured the migration of Hepa1-6 cells treated with different CMs from si-β-catenin (S1)- or siR-transfected M2 macrophages by scratch wound assay. The results showed that si-β-catenin (S1)-transfected M2 macrophages induced a remarkably slower migration than the control and IL-4 alone group after being cocultured for 16 h (Fig. 4d, e). Third, we also detected the migration and invasion of tumor cells using Transwell inserts without Matrigel or with Matrigel through co-culture experiment for 24 or 48 h. The results showed that si-β-catenin(S1)-transfected M2 macrophages significantly limited the numbers of migrated cells (Fig. 4f upper panels; Fig. 4g left panel), as well as the number of invaded cells (Fig. 4f lower panels; Fig. 4g right panel). Finally, we observed the percentage of proliferating CD8^+^ cytotoxic T lymphocytes after coculture with si-β-catenin(S1)- or siR-transfected differentially polarized macrophages by a mixed lymphocyte reaction assay. The FACS assay showed that si-β-catenin(S1)-transfected M0 or M2 macrophages promoted CD8^+^ T-cell proliferation more strongly than siR-transfected M0 or M2 macrophages (Fig. 4h, i). Taken together, these results indicated that knockdown of β-catenin in M2 macrophages abrogated TAM phenotypes in vitro, namely reduced tumor cell proliferation, migration, and invasion and increased the CD8^+^ T-cell number.

### Wnt ligands secreted from tumor cells initiated Wnt/β-catenin signaling activation in M2 macrophages through a paracrine manner

**Fig. 5:**
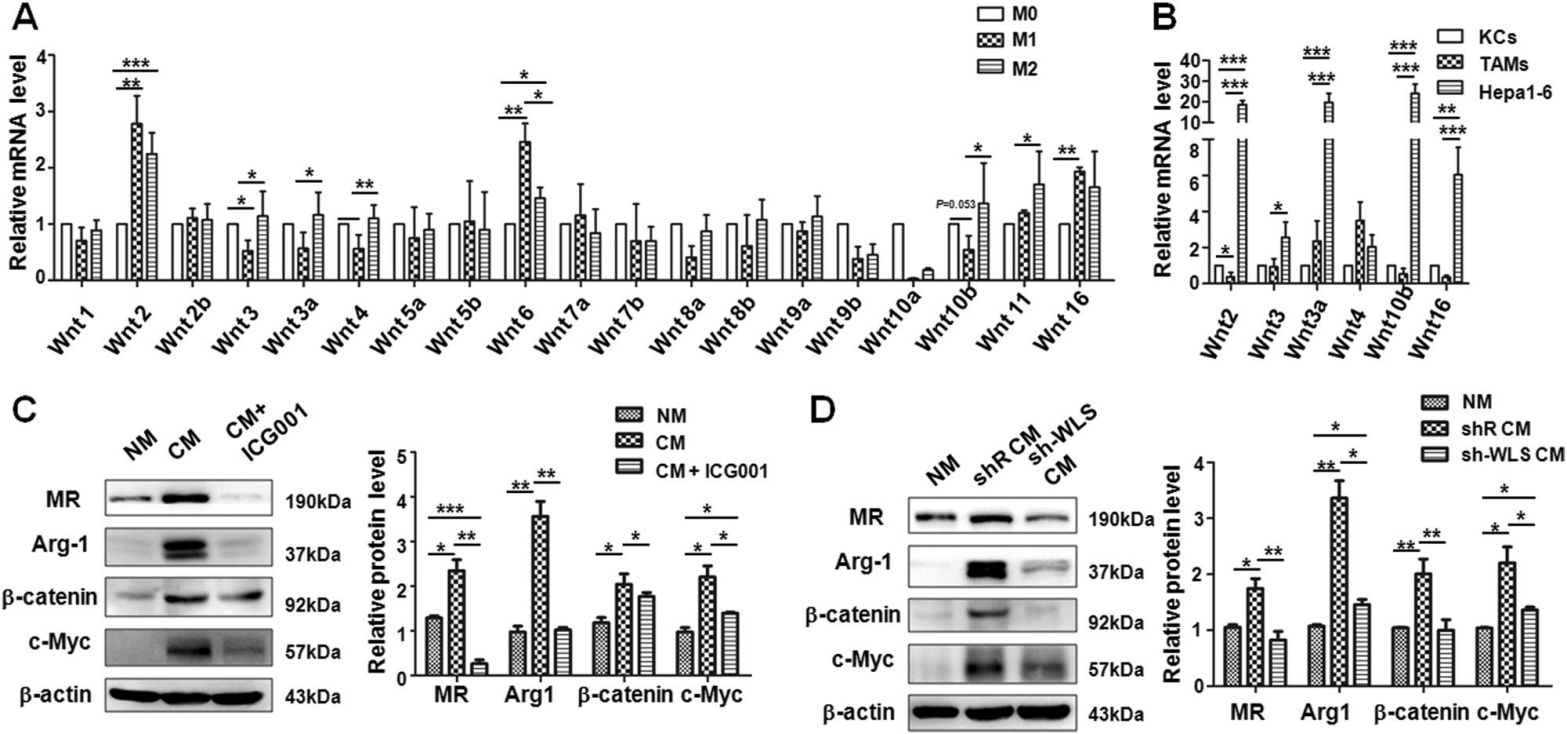
Wnt ligands are secreted by tumor cells cause the Wnt/β-catenin activation in macrophages leading to M2 macrophage polarization. **a** BMDMs (M0) were stimulated with LPS + IFN-γ (M1) or IL-4 (M2). The expression of Wnt ligands was determined by qRT-PCR, using β-actin as an internal control. A quantitative comparison among M0, M1, and M2 macrophages was presented (*n* = 5). **b** The expression of the indicated Wnt ligands in KCs, TAMs, and Hepa1-6 cells was determined by qRT-PCR, and compared after calibration of their β-actin levels (*n* = 3). **c** BMDMs were cultured with the conditional medium (CM) that was collected from cultured Hepa1-6 cells for 24 h. Meanwhile BMDMs were treated with or without ICG001. The M2-related markers and downstream genes of Wnt signaling were detected by western blotting (left panel). The relative protein levels were quantitatively compared (right panel) (*n* = 4). **d** BMDMs were co-cultured with the conditional medium from Hepa1-6 cells that were infected with shRNA targeting the Wntless by lentivirus delivery system for 24 h. The M2-related markers and downstream genes of Wnt signaling were detected by western blotting (left panel). The relative protein levels were quantitatively compared (right panel) (*n* = 3). Bars, mean ± SD; *P < 0.05; **P < 0.01; ***P < 0.001

Because Wnt ligands are expressed by different hepatic cell types including hepatocytes^23^, we postulated that activated Wnt/β-catenin signaling in TAMs might be initiated by Wnt ligands secreted from Hepa1-6 cells in TME. To confirm this possibility, we first examined the mRNA levels of Wnt ligands in differentially polarized macrophage, which showed that the expression of several Wnt ligands, such as Wnt3, 3a, 4, 10b, and 11, were relatively higher in M2 macrophages than that in M1 macrophages, and the expression of Wnt2, 6, 11, and 16 were relatively higher in M1 macrophages compared with that in M0 macrophages (Fig. 5a). Then, the mRNA levels of Wnt2, 3, 3a, 4, 10b, and 16 were compared among KCs, TAMs, and Hepa1-6 cells by qRT-PCR after calibrating their β-actin expression. The result showed that the mRNA level of Wnt ligands was higher in Hepa1-6 cells than that in KCs or TAMs (Fig. 5b). These results suggested that tumor-derived Wnt ligands might trigger Wnt signaling activation in neighboring macrophages through paracrine manner. Next, mature BMDMs were cultured in normal culture medium (NM) or CM from cultured Hepa1-6 cells and treated with or without ICG001 for 24 h. Then, the protein levels of M2 macrophage-related molecules, such as MR and Arg1, and downstream genes of Wnt/β-catenin signaling including β-catenin and c-Myc, were detected by western blotting. The result showed that the expression levels of MR, Arg1, β-catenin, and c-Myc were all increased significantly in macrophages cultured with Hepa1-6 CM, but this effect was reversed after blocking Wnt/β-catenin signaling with ICG001 (Fig. 5c). These data further indicated that activated Wnt/β-catenin signaling in M2 macrophages might be caused by tumor cell-derived Wnt ligands through a paracrine manner.

Wntless (WLS) is an evolutionarily conserved multipass transmembrane protein that is specially required for Wnt ligands secretion^33^. In order to further confirm the induction of M2 macrophages by paracrine Wnt ligands from Hepa1-6 cells, we constructed a lentiviral vector expressing Wntless shRNA. The efficiency of Wntless knockdown in Hepa1-6 cells was confirmed by qRT-PCR (Supplement Fig. 2C). The secreted Wnt3a in the cultured supernatant of Wntless-silenced Hepa1-6 cells was reduced by approximately 60% compared with the shR-infected group as determined by ELISA (Supplement Fig. 2D). Next, we tested macrophage polarization and Wnt/β-catenin signal activation in BMDMs using a co-culture system, and found that the protein level of M2 macrophage-related markers, MR and Arg1, was dramatically decreased in BMDMs that were cultured with CM from sh-WLS-infected Hepa1-6 cells, as compared with that in BMDMs cultured with CM from shR-infected Hepa1-6 cells. The expression of downstream molecules of Wnt/β-catenin signaling including β-catenin and c-Myc was decreased significantly in BMDMs cultured with CM from sh-WLS-infected Hepa1-6 cells (Fig. 5d). Collectively, these results verified that tumor cells stimulated Wnt/β-catenin signal activation in M2-like macrophages through a paracrine manner.

### Blockade of Wnt protein secretion in hepatic tumor cells inhibited hepatic tumor growth by regulating the tumor immune microenvironment

**Fig. 6:**
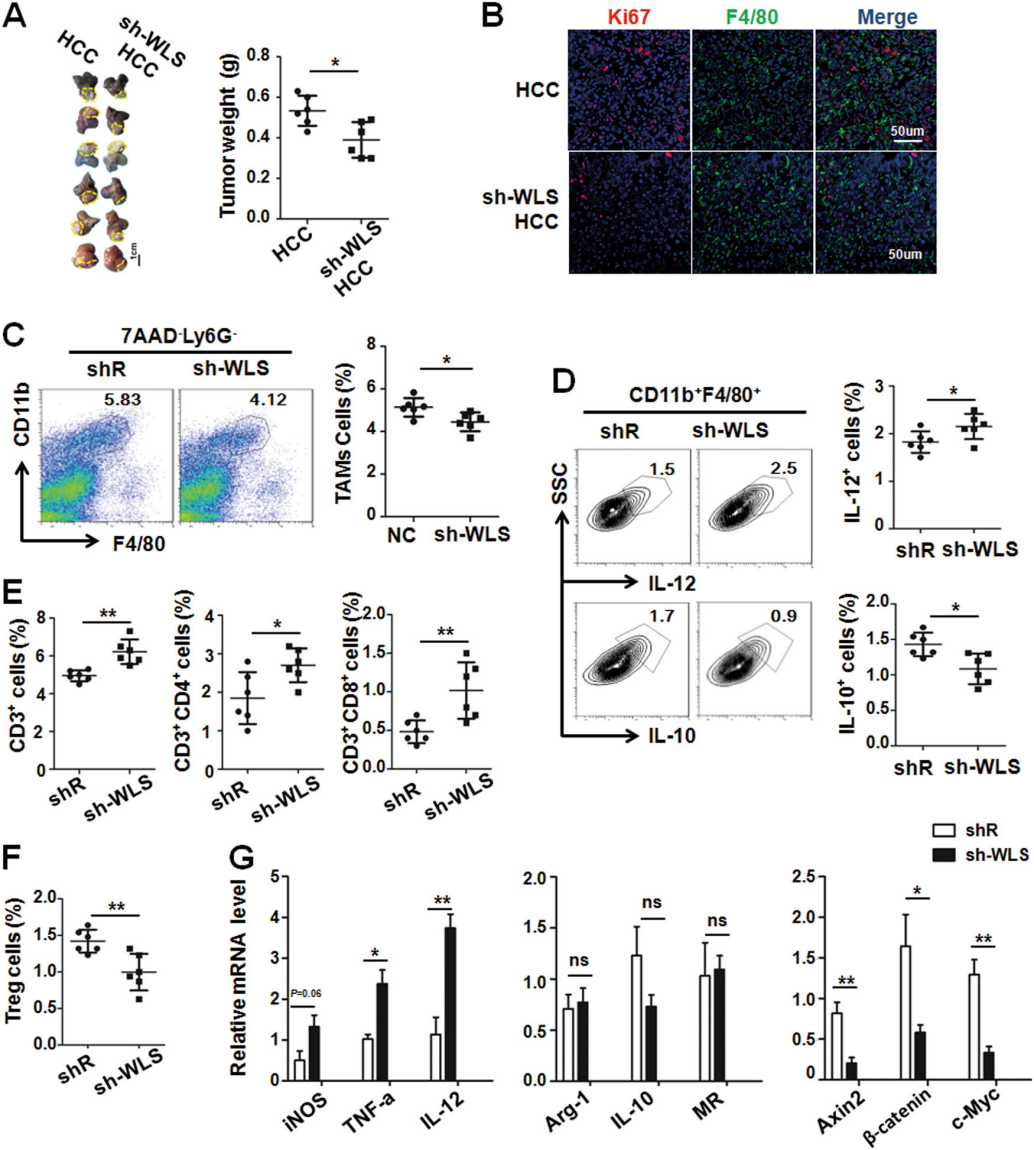
Knockdown Wntless in Hepa1-6 cells inhibited tumor growth by reducing M2-like TAMs and increasing antitumor T cells. **a** Infected Hepa1-6 cells as described in Supplement Fig. 2C were orthotopically inoculated in the liver of C57BL/6 mice. Tumors were dissected 3 weeks after inoculation and photographed (left panel). Tumor weight was compared (right panel) (*n* = 6). **b** Tumor sections were stained with anti-F4/80 and Ki67 using immunofluoresecence staining. Ki67^+^ cells were counted and compared (*n* = 3). **c** Single cells suspensions from tumor tissues in (**a**) were prepared and stained. The Ly6G^-^F4/80^+^ CD11b^+^cells (TAMs) were analyzed by FACS. The percentage of TAMs cells in tumor tissue were compared (*n* = 6). **d** Tumor single cell suspension was analyzed by FACS for detecting the cytoplasmic IL-10 and IL-12 expression in TAMs. The percentage of IL-12^+^ TAMs and IL-10^+^TAMs was compared (*n* = 6). **e** The percentage of CD3^+^ cells, CD3^+^CD4^+^cells and CD3^+^CD8^+^ cells in tumor cell suspensions were analyzed by FACS and then compared (*n* = 6). **f** The percentage of CD4^+^FoxP3^+^CD25^+^ cells (Treg) cells was analyzed by FACS and then compared (*n* = 6). **g** TAMs were sorted from liver of hepatic tumor-bearing mice or sh-WLS infected hepatic tumor-bearing mice, and then the M1/M2-polarized markers and the downstream genes of Wnt/β-catenin signaling were determined by qRT-PCR and quantitatively compared (*n* = 4). Bars, mean ± SD; *P < 0.05; **P < 0.01

Next, we observed the crosstalk between tumor cells and TAMs via Wnt/β-catenin signaling in HCC-bearing mice using orthotopical inoculation of sh-WLS-infected or shR-infected Hepa1-6 cells. After 3 weeks inoculation, mice were sacrificed, and tumors were photographed and measured. As shown in Fig. 6a, knockdown of Wntless in Hepa1-6 cells resulted in retarded growth of orthotopical HCC. Moreover, the proliferation of tumor cells but not F4/80^+^ TAMs in sh-WLS HCC-bearing mice decreased by Ki67 immunofluoresence staining of tumor sections (Fig. 6b). Furthermore, we analyzed tumor-infiltrating immune cells by FACS. As shown in Fig. 6c, the percentage of TAMs was reduced significantly in sh-WLS HCC-bearing mice, and the phenotype of TAMs intended to be M1-like macrophages as determined by FACS after intracellular IL-10 and IL-12 staining (Fig. 6d). This result was similar to the result of β-catenin knockdown in M2 macrophages (Supplement Fig. 4A and B), indicating that blockade of Wnt signal activation in TAMs might reprogram the TAM phenotype from protumor to antitumor. In addition, the percentage of CD3^+^ cells (including CD4^+^ and CD8^+^ T cells) increased while that of CD25^+^Foxp3^+^ Treg cells decreased (Fig. 6e, f). In TAMs isolated from mice bearing sh-WLS-transfected Hepa1-6 tumors, the expression of M1 markers increased accompanied by reduced Wnt/β-catenin signaling, although M2 markers appeared not changed, as determined by qRT-PCR (Fig. 6g). Collectively, these data suggest that blockade of Wnt secretion from tumor cells also changed the tumor immunosuppressive environment.

### Nuclear accumulation of β-catenin is positively correlated with M2-like TAMs in human HCC biopsies

**Fig. 7:**
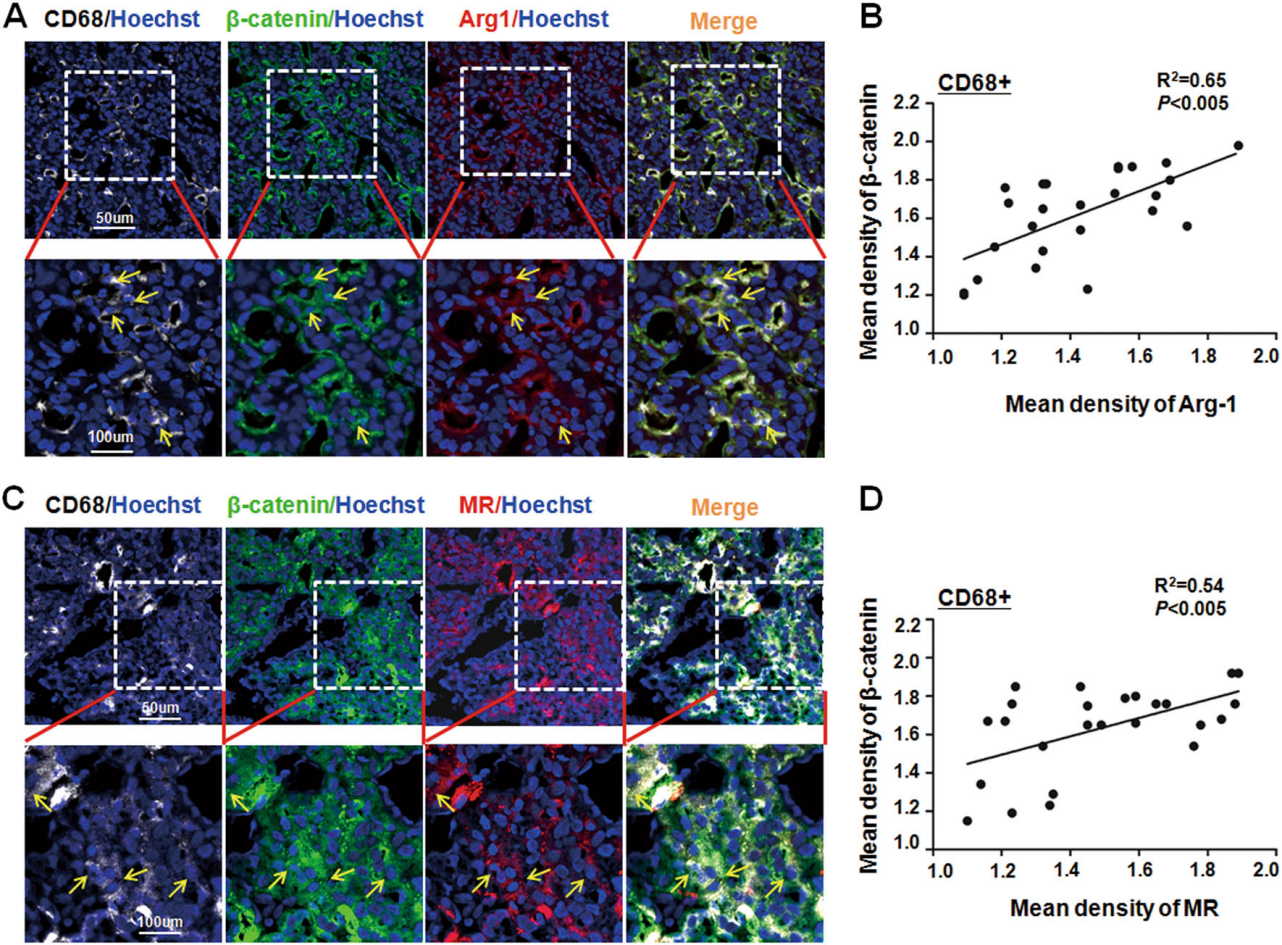
The nucleic accumulation of β-catenin was positively correlated with CD68^+^ TAMs in HCC patient biopsies. **a** Human HCC biopsies were sectioned and stained with anti-CD68 (Cy5), anti-β-catenin (FITC), and anti-Arg1 (Cy3) using immunofluorescence staining, and counterstained with Hoechst. The representative images of CD68^+^β-catenin^+^Arg1^+^ cells in HCC sections were observed (upper panel) and shown with high magnitude (lower panel) (*n* = 25). **b** The expression of β-catenin and Arg1 in randomly selected CD68^+^ cells in (**a**) were quantified using Mean Density (IOD/area). The correlation of the nucleic β-catenin expression with Arg-1^+^CD68^+^ TAMs in HCC patients was analyzed. **c** Human HCC biopsies were sectioned and stained with anti-CD68 (Cy5), anti-β-catenin (FITC), and anti-MR (Cy3) using immunofluorescence staining, and counterstained with Hoechst. The representative images of CD68^+^β-catenin^+^MR^+^ cells in HCC sections were observed (upper panel) and shown with high magnitude (lower panel) (*n* = 25). **d** The expression of β-catenin and MR in randomly selected CD68^+^ cells in (**c**) were quantified using Mean Density (IOD/area). The correlation of the nucleic β-catenin expression with MR^+^CD68^+^ TAMs in HCC patients was analyzed

The role of TAMs in tumorigenesis has been reported in many different solid tumors^3^. To further dissect the relationship between activated Wnt/β-catenin signaling and M2 macrophages in HCC patients, we determined the expression of CD68 (a pan macrophage marker), β-catenin and M2-related markers MR or Arg1 in 25 frozen tumor sections by immunofluorescence staining. As shown in Fig. 7a, c, many CD68^+^Arg1^+^ or CD68^+^MR^+^ M2-like TAMs infiltrated into HCC tumors and some of them exhibited an obvious nuclear localization of β-catenin. Data quantification showed that the level of nuclear β-catenin was positively correlated with the Arg1 or MR expression in CD68^+^ macrophages in HCC patient biopsies (Fig. 7b, d). Therefore, these results further indicated that activated Wnt/β-catenin signaling is involved in M2-like TAMs during HCC tumorigenesis.

## Discussion

Wnt signaling is an evolutionarily conserved pathway involved in embryonic development and tissue homeostasis maintenance. Aberrant mutations in components of Wnt signaling have been linked to multiple growth-related pathologies and cancer, particularly to HCC^14,16,34^. Wnt ligands are secreted protein that can mediate cell–cell interactions by a paracrine manner. In chronic liver disease, macrophage-derived Wnt3a induces Wnt/β-cantenin activation in hepatic progenitor cells and promotes their specification to hepatocytes^24^. Recently, several studies have reported that Wnt ligands can be synthesized by macrophages leading to Wnt signaling activation in tumor cells^20–22^. Conversely, whether Wnt ligands secreted from tumor cells can promote Wnt signal activation in macrophages is still elusive. In our study, we show that HCC tumor cells-derived Wnt ligands activate the canonical Wnt/β-catenin signaling of macrophages and then promote them to polarize to M2-like TAMs by a paracrine mode. Moreover, blocking the expression of β-catenin in BMDMs in the presence of IL-4 reduces Hepa1-6 cell proliferation, migration and invasion, as well as enhances CD8^+^ T-cell proliferation (Supplement Fig. 5). However, we also find that activated macrophages express some Wnt ligands (Fig. 5a), whether Wnt/β-catenin signaling can regulate macrophage polarization in an autocrine manner in vivo needs further study.

β-catenin is a crucial component of canonical Wnt signaling. In the non-activated state, the cytoplasmic β-catenin level remains low due to an interaction with a destruction complex that is composed of APC, Axins, CK1α, and GSK3β. In the presence of canonical Wnt ligands, this destruction complex is inactivated and then allows the cytoplasmic β-catenin to translocate into the nucleus and combine with the transcription factors LEF and TCF leading to the transcription of target genes of including c-Myc, cyclin D1, and Axin2^14^. In our study, we find that the expression of β-catenin, c-Myc and Axin2 increases in M2 macrophages but not in M1 macrophages. Moreover, Wnt3a or LiCl treatment boosted macrophages to polarize to M2 macrophages under an inflammatory stimulus, and knockdown of β-catenin in M2 macrophages abolishes the functions of TAMs when co-cultured with tumor cells. These results indicate that canonical Wnt/β-catenin signal activation in macrophages promotes them to adopt the phenotype of M2-like TAMs, which play a critical role in tumor progression. Similarly, Feng et al. reported that activation of Wnt/β-catenin signaling promotes kidney fibrosis by stimulating M2-polarized macrophages^35^. In addition, in our current work, we also observe that Wnt4 and Wnt5a are both expressed by hepatocytes and macrophages^23^^,^^36^, which can mediate noncanonical Wnt/Ca2^+^ signaling. Therefore, in addition to Wnt3a-triggered Wnt/β-cantenin signaling activation in macrophages, whether noncanonical Wnt signaling can be involved in the regulation of macrophage polarization and function still needs more exploration.

c-Myc has been studied mostly in tumor cells as a proto-oncogene^37^. Recently, its function in nontumoral immune cells has been gradually disclosed. c-Myc not only participates in hematopoietic stem cell development and differentiation but also plays a role in the B lymphocyte homeostasis and survival/apoptosis of myeloid cells^38,39,40,41^. Moreover, Pello et al. finds that c-Myc is expressed in TAMs of human colon cancer and is responsible for M2 macrophage activation^31,42^. Recently, Hadjidaniel et al. reported that c-Myc in neuroblastomas induces TAM activation and phenotype depending on STAT3 phosphorylation in the absence of IL-6^43^. Because c-Myc is also a key downstream gene of Wnt/β-cantenin signaling, in the present study, we report that this transcription factor is required for macrophage alternative activation when Wnt/β-cantenin signaling is activated in macrophages.

In liver development and tumorigenesis, Wnt signaling and Notch signaling are mutually regulated^44,45^. Our previous studies have demonstrated that Notch signaling is required for macrophage M1 polarization in inflammatory state^46,47,48^, and activated Notch signaling in macrophages promotes hepatic fibrosis by upregulation of NF-κB through CYLD^49^. Because M2 polarization can be thought as a “default” state of Notch signaling, the literature and our current study all show that activated Wnt/β-cantenin signaling is involved in M2 macrophage polarization^35^. Therefore, it is interesting to ask whether there is a negative correlation between Notch and Wnt signaling in macrophage polarization and function during tumorigenesis. In the future, we will address this question by using macrophage-specific Notch or Wnt knockout mice in combination with an HCC model.

In summary, our study is the first to demonstrate that tumor-derived Wnt ligands can activate canonical Wnt/β-cantenin signaling in macrophages and cause macrophage M2 polarization, resulting in tumor growth, migration, and metastasis. Blockade of Wnt secretion from tumor cells or Wnt signaling activation in M2-like TAMs should be a potential strategy for HCC therapy in the future.

## Materials and methods

### Patient material

Tissues of HCC were collected from 25 patients who received an operation in Department of Hepatobiliary Surgery, Xijing Hospital, Fourth Military Medical University during August 2016 to April 2017. The study was approved by the Ethical Committee of Xijing Hospital and informed consent was obtained from all patients. The clinical data of all patients involved in this study are presented in Supplement Table 1.

### Mice and liver tumor model

C57BL/6 mice (8–10 weeks of age) were maintained in a specific pathogen-free (SPF) environment. The liver tumor model was established using C57BL/6 mice as follows: for the orthotropic HCC model, Hepa1-6 cells (5.0 × 10^6^) in 30 μl Matrigel (Corning, NY, USA) were orthotopically inoculated into the left liver lobes of mice under anesthesia with 0.6% pentobarbital sodium solution (10 µL/g, Sigma, Louis, Missouri, USA). After 3 weeks, mice were sacrificed. Tumor weight was measured. And then tumor was minced for further analysis. All mouse experiments were conducted according to a guideline from the Animal Experiment Administration Committee of the Fourth Military Medical University and the Guide for the Care and Use of Laboratory Animals published by the National Institutes of Health (NIH publication 86-23, revised 1985).

### Cell culture and transfection

BMDMs were induced and cultured as previously described^47^. Briefly, bone marrow cells were isolated from mouse femurs and tibias, and then were cultured in Dulbecco’s modified Eagle’s medium (DMEM, Gibco, Waltham, MA, USA) containing 10% fetal calf serum (FCS, Gibco) and 25 ng/mL murine macrophage-colony stimulating factor (M-CSF, Sino Biological Inc, Beijing, China) for 7 days to obtain BMDMs. M1 or M2-polarized macrophages were induced by adding LPS (50 ng/mL, Sigma) + rIFN-γ (20 ng/mL, PeproTech, Rocky Hill, USA) or IL-4 (20 ng/mL, PeproTech) into the cultured BMDMs for 24 h, respectively. In some experiments, after being treated with Wnt activator (Wnt3a, RD, Minneapolis, USA), BMDMs were stimulated with LPS + rIFN-γ or IL-4 for further analysis. For transfection, cultured BMDMs were transfected with 100 nM β-catenin or c-Myc small interfering RNA (siRNA-β-catenin or siRNA-c-Myc) or randomized siRNA control (siR) (Ribo Bio, Guangzhou, China) using the Lipofectamine LTX (Invitrogen, Grand Island, NY, USA) according to the protocol. Total RNA or proteins were extracted 48 h after the transfection. All siRNA sequences are shown in Supplement Table 2.

The murine hepatoma cell line (Hepa1-6) was originally purchased from ATCC (American Type Culture Collection, No. CRL-1830™). Cells were cultured in DMEM supplemented with 10% FCS, 2 mM l-glutamine (Gibco) and 1% penicillin/streptomycin (Gibco) at 37 °C in a 95% air-5% CO_2_ incubator. In some cases, the CM from cultured Hepa1-6 cells was collected and added into BMDMs to induce M2-like macrophages for 24 h. Meanwhile, the inhibitor of Wnt signaling, ICG001 (MCE, Monmouth Junction, USA), was supplied into the medium. Then, cells were collected for further experiments.

### The colony-forming assay

Hepa1-6 cells were seeded into six-well plate at a density of 200 cells per well and cultured with CM, which was collected from cultured M2 macrophages transfected with si-β-catenin or randomized siRNA control (siR) for 2 weeks. After that, Hepa1-6 cells were washed with PBS twice, fixed with 4% paraformaldehyde (Sigma) and stained with 1% crystal violet (LEAGENE, Beijing, China). The number of colonies was counted.

### Scratch wound assay

Hepa1-6 cells were seeded into 12-well plates at a density of 2 × 10^6^/well and grown to 80–90% confluence. The cells were scratched with a sterile 200 µL micropipette tip and the debris were washed away. Then the cells were cultured with CM as described above for 0, 8, and 16 h. The cell migration distance between the two scratches was recorded and measured with ImageJ software. The cell migration distance was calculated as follows: average gap area at 0 h – average gap area at 8/16 h.

### Migration and invasion assays

BMDMs were seeded into the 12-well plate and transfected with si-β-catenin or siR as described above. And then BMDMs were stimulated with IL-4 for 24 h. Meanwhile, 4 × 10^4^ Hepa1-6 cells were suspended in 200 µL serum-free DMEM and plated in upper chamber without Matrigel (for the migration experiment) or with Matrigel (for the invasion experiment) coated (Corning). After incubating at 37 °C for 24 h (for the migration experiment) or 48 h (for the invasion experiment), the non-invading cells were scrubbed away by a cotton swab. The cells that had penetrated through the filter were stained with crystal violet and counted under a microscope. Ten randomly selected fields were used to count the number of migrating cell number in each insert.

### Mixed lymphocyte reaction

A mixed lymphocyte reaction was performed as previously described^48^. BMDMs were seeded into 24-well plates. After transfection with si-β-catenin or siR, macrophages were stimulated with PBS (M0), LPS + INF-γ (M1) or IL-4 (M2) for 24 h. Then, cells were irradiated with 20 Gy X-rays. Naïve T cells were negatively sorted from the lymph nodes of allogeneic mice using biotin-labeled B cell- and myeloid cell-specific antibodies, followed by magnetic Streptavidin Particles Plus (BD IMag^TM^, Franklin Lake, New Jersey, USA). T cells were labeled with carboxyfluorescein diacetatesuccinimidyl ester (CFSE, Invitrogen) and added into irradiated differentially polarized BMDMs at a ratio of 5:1.5 days later, nonadherent cells were harvested and stained with APC anti-CD8 antibody (BD Pharmingen). Proliferation of CD8^+^ T cells was analyzed by FACS.

### Flow cytometry

Tumors tissues were dissected from PBS-perfused mice. After measuring their weight, tumors were finely chopped and incubated with 1 mg/mL collagenase type V (Sigma) and 100 µg/mL DNase I (Roche, Basel, Switzerland) for 1 h at 37 °C. Digested tissues were mashed through cell strainers (70 µm) and subjected to removal of red blood cells. The harvested single cells were incubated with relevant antibody cocktails for 20 min on ice in the dark following the standard protocols. Biotin-labeled primary antibodies were detected with PE-streptavidin as the secondary antibody. Cell viability was evaluated using 7-AAD (BD Biosciences). For intracellular staining, cells were permeabilized with Permeabilization Buffer (Invitrogen) and stained with PE-conjugated primary antibodies. FACSCalibur^TM^ flow cytometer (BD Immunocytometry Systems) were applied for sample analysis. FACS data were analyzed using Flowjo vX.0.6 software (TreeStar, Ashland, OR).

The primary Kuppfer cells (KC) from the liver of wild-type mice or TAMs from orthotopic HCC-bearing mice were sorted using a FACSAriaII flow cytometer (BD Biosciences). The sorted cells were subjected to preparation of total mRNA for subsequent analysis of gene expression. The information of all the antibodies used is shown in Supplement Table 3.

### qRT-PCR

Total RNA of cells was extracted using TRIzol (Invitrogen) or LS TRIzol reagent (Invitrogen) according to the manufacturer’s instruction. Reverse transcription was performed using a kit with random primers (Takara, Dalian, China), and then real-time PCR was carried out using QuantiTect SYBR Green PCR Kit (Takara) on the ABI PRISM 7500 real-time PCR system (Applied Biosystems), with β-actin as an internal control. All the primers used are shown in Supplement Table 2.

### Immunofluorescence

For cell immunofluorescence, cultured cells were grown on cover slides until confluence, and fixed in 4% paraformaldehyde for 15 min, followed by three washes with PBS. Cells were incubated with primary antibodies including anti-F4/80, anti-β-catenin, anti-MR, anti-Arg1 or Ki67 followed by secondary antibody staining. Nuclei were counterstained with Hoechst 33258 (Sigma). Photographs were taken using a laser scanning confocal microscope (FV1000, Olympus).

Liver sections from biopsies of HCC patients were prepared according to standard procedures. The primary antibodies included anti-CD68, anti-β-catenin, anti-MR, or anti-Arg1 followed by secondary antibodies staining. All antibodies used are listed in Supplement Table 3.

### Western blotting

Western blotting was performed according to standard procedure. Each 40 µg protein samples were loaded and separated by SDS-PAGE, and subsequently transferred onto polyvinylidene fluoride membranes. The protein-blotted membranes were incubated with primary antibodies overnight at 4 °C. HRP-conjugated secondary antibodies were incubated at room temperature for 1 h. The chemoluminescence signals were developed with Pierce ECL Western Blotting Substrate (Thermo Scientific, Waltham, MA, USA) and detected using the ChemiScope imaging system (Clinx Science, Shanghai, China). The protein expression level was quantified and normalized to β-actin as an internal reference with ImageJ software (NIH). The information of all antibodies used is listed in Supplement Table 3.

### Enzyme-linked immunosorbent assay (ELISA)

The shRNA targeting the Wntless (sh-Wntless) and the control shRNA were inserted into the lentiviral expression vector (GV493, GENECHEM). In addition, viral particles were prepared by transfecting HEK293T. Hepa1-6 cells were transduced following the recommended protocols. The shRNA sequence against Wntless was shown in Supplement Table 2. The efficiency of Wntless knockdown in Hepa1-6 cells was confirmed by RT-PCR and ELISA. The supernatant was collected from cultured Hepa1-6 cells that had been transduced with sh-Wntless successfully, and then the concentration of Wnt3a in the supernatant was detected using a mouse Wnt3a ELISA kit (MyBioSource, California, USA) according to the instructions.

### Statistics

All the images were quantified by Image Pro Plus 5.1 software (Media Cybernetics Inc.). Data were analyzed using Graph Pad Prism 5 software (San Diego). An unpaired Student’s *t* test, paired *t* test or one-way ANOVA with Tukey’s multiple comparison test was used for the statistical analysis. *P* < 0.05 was considered statistically significant.

## Electronic supplementary material

Supplement Material
