# Supplementary material for: Crosstalk between hepatic tumor cells and macrophages via Wnt/β-catenin signaling promotes M2-like macrophage polarization and reinforces tumor malignant behaviors
Source: Cell Death Dis. 2018 Jul 18;9(8):793. doi: 10.1038/s41419-018-0818-0 (PMC6052107; doi:10.1038/s41419-018-0818-0)
Supplement: Supplementary file 1 — Supplement Material [file 41419_2018_818_MOESM1_ESM.docx]

**Supplementary Material**


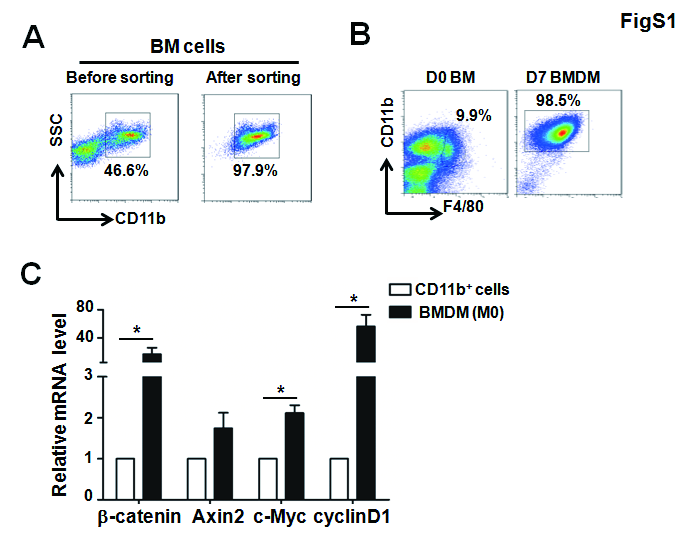


**FigS1. Wnt/β-catenin signaling is activated during the differentiation of monocytes into macrophages. (A)** CD11b^+^ monocytes were sorted from bone marrow (BM) cells of C57BL/6 mice by MACS. The cell purity was detected by FACS using anti-CD11b antibody. Dead cells were excluded by 7AAD staining (n=3). **(B)** BM cells were cultured in the presence of M-CSF for 7days to obtain BM-derived macrophages (BMDMs). BMDMs phenotype was analyzed by FACS with antibodies for F4/80 and CD11b (n=3). **(C)** The relative level of downstream genes of Wnt /β-catenin signaling in CD11b+ monocytes and BMDMs was determined by qRT-PCR (n=3 ). Bars, mean ± SD; *P < 0.05.


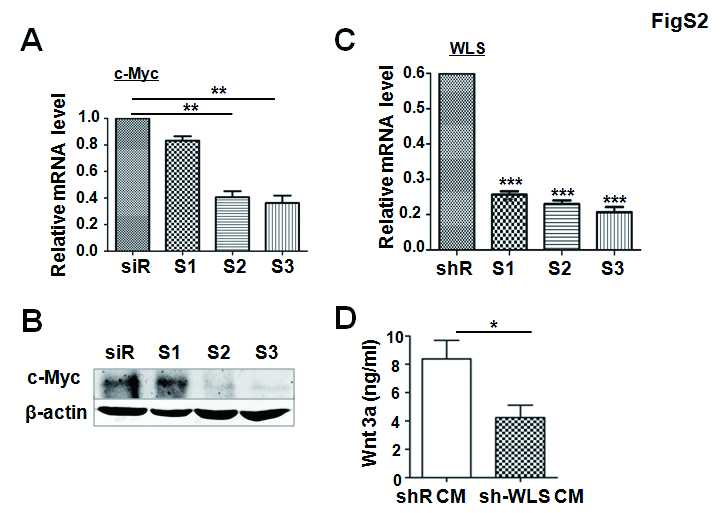


**FigS2. Knockdown of c-Myc in BMDMs and knockdown of Wntless in Hepa1-6 cells. (A-B)** BMDMs were transfected with c-Myc siRNA (S1, S2 or S3) or control oligo (siR) for 24 h. The expression level of c-Myc in BMDMs was determined by qRT-PCR (A) and Western bloting (B) (n=3). **(C)** Hepa1-6 cells were infected with shRNA targeting the Wntless (S1, S2 or S3) or the control (shR) by lentivirus delivery system. The mRNA level of Wntless was determined by qRT-PCR (n=3). **(D)** The protein level of Wnt3a in cultured supernatants of infected Hepa1-6 cells as (C), was examined by ELISA (n=3). Bars, mean ± SD; *P < 0.05; **P < 0.01; ***P < 0.001.


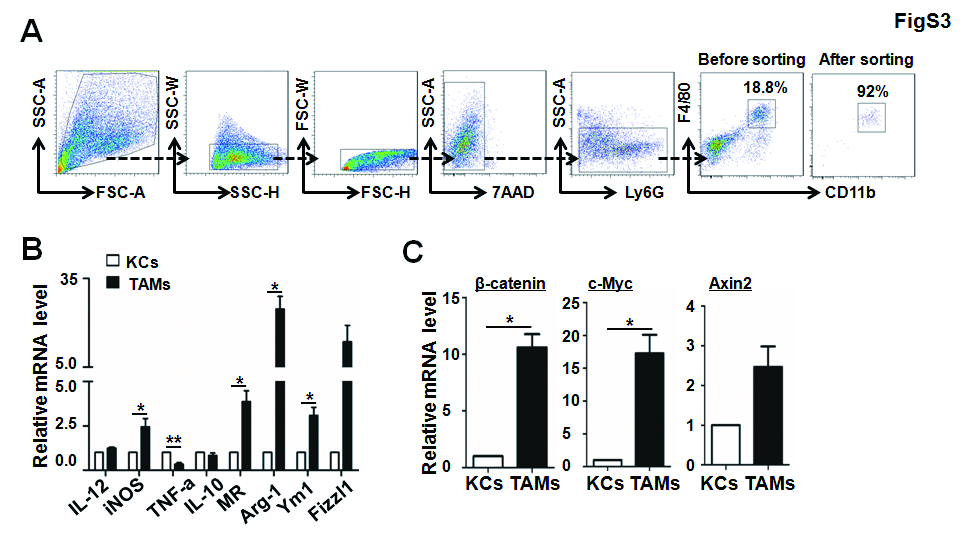


**FigS3. Wnt/β-catenin signaling is activated in tumor-associated macrophages of hepatic hepa1-6-bearing mice. (A)** The tumor cells suspension were isolated from hepa1-6-bearing mice, and then analyzed and sorted by FACSAriaII after anti-mouse F4/80, anti-Ly6G and anti-CD11b staining. The Ly6G^-^F4/80^+^CD11b^+^ cells in tumor represented TAMs. The cell purity was further analyzed by FACS after sorting. **(B, C)** Kuppfer cells (KCs) were sorted from liver of normal mice as described in A. The expression of M1 and M2 markers (B) and the down-stream genes of Wnt/β-catenin Signaling (C) were detected by qRT-PCR, and then were quantitatively compared (n=3). Bars, mean ± SD; *P < 0.05.


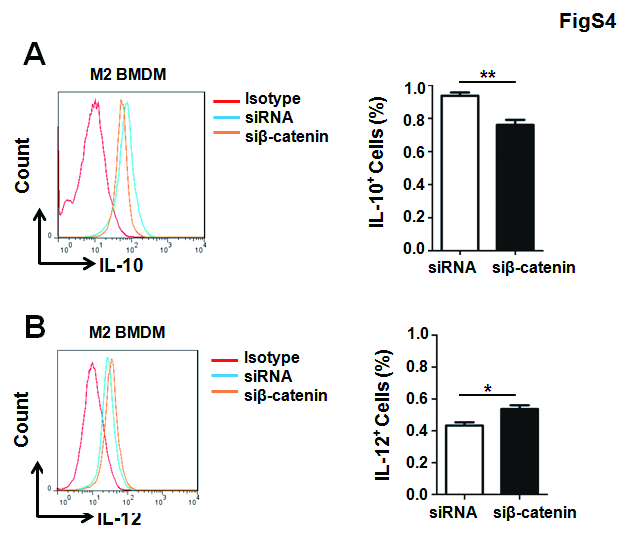


**FigS4. Knockdown β-catenin in M2 BMDMs promoted M2 BMDMs switching to M1 BMDMs.** BMDMs were transfected with β-catenin siRNA or siR followed by IL-4 stimulation for 24 h. The cells were collected and stained with anti-F4/80 and anti-CD11b antibodies, and then treated for cytoplasmic staining with anti-IL-10 or anti-IL-12 antibody, further analyzed by FACS. **(A)** The expression of IL-10 in M2 BMDMs was analyzed and compared (n=3). **(B)** The expression of IL-12 in M2 BMDMs was analyzed and compared (n=3). Bars, mean ± SD; *P < 0.05; **P < 0.01.


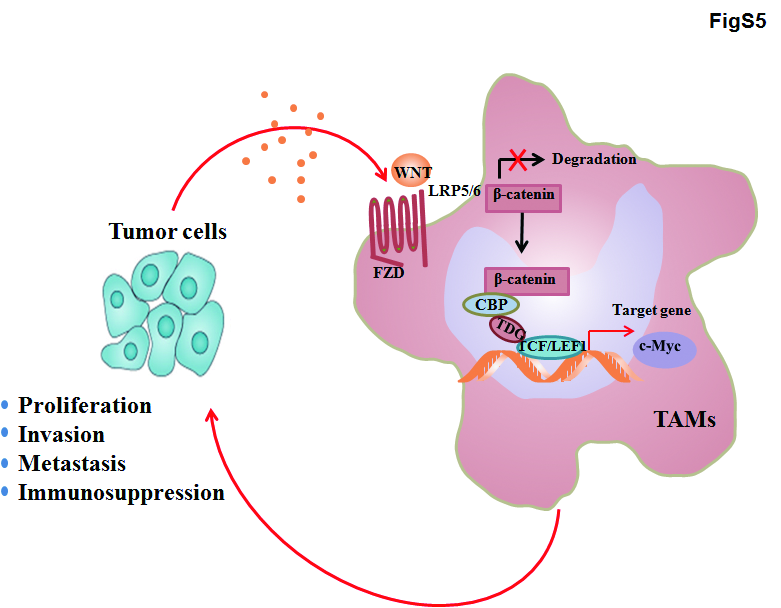


**FigS5. Schematic of Canonical Wnt/β-Catenin signaling promotes M2-Like TAMs during tumor progression.** Wnt ligands, such as Wnt3a, were secreted by Hepatocellular carcinoma (HCC), and then activated the Wnt/β-catenin signaling in macrophages which promoted macrophages polarized to M2-like tumor-associated macrophages (TAMs) through c-Myc by paracrine manner. Consequently, activated Wnt/β-catenin signaling in M2-like TAMs promoted HCC growth, invasion, metastasis and immune suppression.

**Supplement Table 1.**Clinical data of 25 HCC patients involved in this study.

| **No.** | **Sex** | **Age** | **AJCCstage**  **( T stage )** |
| --- | --- | --- | --- |
| 1 | M | 52 | T2 |
| 2 | M | 40 | T1 |
| 3 | M | 46 | T2 |
| 4 | F | 66 | T2 |
| 5 | M | 38 | T2 |
| 6 | F | 64 | T2 |
| 7 | M | 51 | T2 |
| 8 | M | 50 | T1 |
| 9 | M | 62 | T1 |
| 10 | M | 59 | T1 |
| 11 | F | 63 | T1 |
| 12 | M | 46 | T1 |
| 13 | M | 54 | T2 |
| 14 | M | 63 | T2 |
| 15 | M | 51 | T1 |
| 16 | M | 48 | T2 |
| 17 | F | 45 | T1 |
| 18 | M | 65 | T2 |
| 19 | M | 49 | T1 |
| 20 | M | 58 | T3 |
| 21 | M | 55 | T3 |
| 22 | F | 59 | T2 |
| 23 | F | 49 | T1 |
| 24 | M | 57 | T3 |
| 25 | M | 62 | T1 |

**Supplement Table 2.**Primers and oligonucleotides used in this study.

| **Gene (Wnt signaling)** | **Purpose** | **Sequence (5’-3’)** |
| --- | --- | --- |
| Wnt1-F | RT-PCR | ATCCATCTCTCCCACCTCCTAC |
| Wnt1-R | RT-PCR | GAATCTTTCTCTCACCCTCTGG |
| Wnt2-F | RT-PCR | GTGATGTGTGACAATGTGCCA |
| Wnt2-R | RT-PCR | GTTGCAGTTCCAGCGATGC |
| Wnt3-F | RT-PCR | AGCGTAGCAGAAGGTGTGAAG |
| Wnt3-R | RT-PCR | CCAGGTGGCCCCTTATGATG |
| Wnt3a-F | RT-PCR | TCGGAGATGGTGGTAGAGAAAC |
| Wnt3aR | RT-PCR | TCGCAGAAGTTGGGTGAGG |
| Wnt4-F | RT-PCR | AGAACTGGAGAAGTGTGGCTGT |
| Wnt4-R | RT-PCR | AAAGGACTGTGAGAAGGCTACG |
| Wnt5a-F | RT-PCR | GTCCTTTGAGATGGGTGGTATC |
| Wnt5aR | RT-PCR | ACCTCTGGGTTAGGGAGTGTCT |
| Wnt5b-F | RT-PCR | TGTCAGTTGTATCAGGAGCACA |
| Wnt5b-R | RT-PCR | GTGAAGGCAGTCTCTCGGCTA |
| Wnt6-F | RT-PCR | TTTACACCAGCCCACGAAAG |
| Wnt6-R | RT-PCR | ACTCACCCATCCATCCCAGTA |
| Wnt7a-F | RT-PCR | GGATGCTCACAGGGAAAGAAC |
| Wnt7a-R | RT-PCR | GCAGGAAACCCAGAATACCC |
| Wnt7b-F | RT-PCR | TGAAGCTGGAATGTAAGTGTCAC |
| Wnt7b-R | RT-PCR | CGCTGCGTTGTACTTCTCCT |
| Wnt8a-F | RT-PCR | ACGGTGGAATTGTCCTGAGCATG |
| Wnt8a-R | RT-PCR | GATGGCAGCAGAGCGGATGG |
| Wnt8b-F | RT-PCR | GTTTGCTTGGGACCGTTG |
| Wnt8b-R | RT-PCR | TCCATTTCGGGAGTCATCA |
| Wnt9a-F | RT-PCR | ATGGTGTGTCTGGCTCCTG |
| Wnt9a-R | RT-PCR | CAGTGGCTTCATTGGTAGTGCT |
| Wnt9b-F | RT-PCR | GGGTGTGTGTGGTGACAATCT |
| Wnt9b-R | RT-PCR | GGTCCTTGCTTCCTCTCTTG |
| Wnt10a-F | RT-PCR | TCCTGTTCTTCCTACTGCTGCT |
| Wnt10a-R | RT-PCR | ACGCACACACACCTCCATC |
| Wnt10b-F | RT-PCR | CCACTACAGCCCAGAACCTC |
| Wnt10b-R | RT-PCR | GGAGAGACCCTTTCAACAACTG |
| Wnt11-F | RT-PCR | CCCTGGAAACGAAGTGTAAATG |
| Wnt11-R | RT-PCR | AGGTAGCGGGTCTTGAGGTC |
| Wnt16-F | RT-PCR | GCTGTAACCTCCTCTGCTGTG |
| Wnt16-R | RT-PCR | GTGGACATCGGTCATACTTTCA |
| Wnt2b-F | RT-PCR | ACCTTCCTCTACCCTCAATCCT |
| Wnt2b-R | RT-PCR | TCACTCAGCCTCCTAAATCCAT |
| Fzd4-F | RT-PCR | CCCTAGCAGTCCATCCCTCG |
| Fzd4-R | RT-PCR | AACTCCGGATAGGGAATGCG |
| Fzd7-F | RT-PCR | AGGCGAGTGGGAGATACAGT |
| Fzd7-R | RT-PCR | GCCTTTCCTCAGGGAGACAC |
| Fzd9-F | RT-PCR | TTGCTCTATTATTTCGGGATGGC |
| Fzd9-R | RT-PCR | CAGGACCACGATAGTTTTGAGTG |
| Axin2-F | RT-PCR | GCTGCGCTTTGATAAGGTCC |
| Axin2-R | RT-PCR | GCAATCGGCTTGGTCTCTCT |
| β-catenin-F | RT-PCR | ATGGA GCCGG ACAGA AAAGC |
| β-catenin-R | RT-PCR | TGGGA GGTGT CAACA TCTTCTT |
| c-Myc-F | RT-PCR | GAC AGT GTT CTC TGC CTC TG |
| c-Myc-R | RT-PCR | TCT GCT GTT GCT GGT GAT AG |
| CyclinD1-F | RT-PCR | CGCCCTCCGTATCTTACTTC |
| CyclinD1-R | RT-PCR | AAGCGGTCCAGGTAGTTCAT |
| Wntless-F | RT-PCR | TATCACCTTGGCTTGTGCTG |
| Wntless-R | RT-PCR | TGGGATGGTGCATACAAGAA |

| **Oligonucleotides** | **Purpose** | **Sequence(5’-3’)** |
| --- | --- | --- |
| β-catenin-siRNA1 | siRNA | GCACCAUGCAGAAUACAAA |
| β-catenin-siRNA2 | siRNA | GAAUGAGACUGCAGAUCUU |
| β-catenin-siRNA3 | siRNA | CAAGCCUUAGUAAACAUAA |
| c-Myc-siRNA1 | siRNA | GCGACGAGGAAGAGAAUUU |
| c-Myc-siRNA2 | siRNA | GGAGAUGAUGACCGAGUUA |
| c-Myc-siRNA3 | siRNA | CCACUCACCAGCACAACUA |
| Wntless-shRNA1 | shRNA | GCACGAAGGTCGTTATTAT |
| Wntless-shRNA2 | shRNA | CAAGGGAAATTGAAGCAAA |
| Wntless-shRNA3 | shRNA | GTGTGAAAGAGGAGTACAA |

| **Gene** | **Purpose** | **Sequence (5’-3’)** |
| --- | --- | --- |
| TNF-a-F | RT-PCR | CAG GAG GGA GAA CAG AAA CTC CA |
| TNF-a-R | RT-PCR | CCT GGT TGG CTG CTT GCT T |
| iNOS-F | RT-PCR | GCAGAGATTGGAGGCCTTGTG |
| iNOS-R | RT-PCR | GGGTTGTTGCTAACTTCCAGTC |
| IL-12-F | RT-PCR | GGA AGC ACG GCA GCA GAA TA |
| IL-12-R | RT-PCR | AAC TTG AGG GAG AAG TAG GAA TGG |
| IL-10-F | RT-PCR | CCC TTT GCT ATG GTG TCC TT |
| IL-10-R | RT-PCR | TGG TTT CTC TTC CCA AGA CC |
| MR-F | RT-PCR | AAA CAC AGA CTG ACC CTT CCC |
| MR-R | RT-PCR | GTT AGT GTA CCG CAC CCT CC |
| Arg-1-F | RT-PCR | AGA CAG CAG AGG AGG TGA AGA G |
| Arg-1-R | RT-PCR | CGA AGC AAG CCA AGG TTA AAG C |
| Ym1-F | RT-PCR | CATTCAGTCAGTTATCAGATTCC |
| Ym1-R | RT-PCR | AGTGAGTAGCAGCCTTGG |
| Fizzl1-F | RT-PCR | TGG AGA ATA AGG TCA AGG AAC |
| Fizzl1-R | RT-PCR | GTC AAC GAG TAA GCA CAG G |
| β-actin-F | RT-PCR | CAT CCG TAA AGA CCT CTA TGC CAA C |
| β-actin-F | RT-PCR | ATG GAG CCA CCG ATC CAC A |

**Supplement Table 3. Antibodies used in this study.**

| **Name** | **Supplier** | **City** | **state/province** | | **Country** | **Titration** |
| --- | --- | --- | --- | --- | --- | --- |
| Alexa488 anti-F4/80 | Biolegend | San Diego | | California | USA | 1:100 |
| Biotin anti-Ly6G | Biolegend | San Diego | | California | USA | 1:1000 |
| PE-streptavidin | eBiosicence | Waltham | | Massachusetts | USA | 1:200 |
| APC anti-CD11b | Biolegend | San Diego | | California | USA | 1:400 |
| FITC anti-CD8 | BD-pharmingen | San Diego | | California | USA | 1:50 |
| APC anti-CD8 | BD-pharmingen | San Diego | | California | USA | 1:100 |
| PE anti-CD4 | BD-pharmingen | San Diego | | California | USA | 1:200 |
| APC anti-CD3 | Biolegend | San Diego | | California | USA | 1:200 |
| PE anti-IL-12 | eBiosicence | Waltham | | Massachusetts | USA | 1:50 |
| PE anti-IL-10 | eBiosicence | Waltham | | Massachusetts | USA | 1:50 |
| FITC anti-CD25 | BD-pharmingen | San Diego | | California | USA | 1:50 |
| PE anti-FoxP3 | eBiosicence | Waltham | | Massachusetts | USA | 1:400 |
| APC anti-CD4 | BD-pharmingen | San Diego | | California | USA | 1:100 |
| 7AAD | BD Biosicences | San Diego | | California | USA | 1:100 |
| Rab anti-β-catenin | Abcam | Cambridge | | Massachusetts | USA | 1:2000(WB)  1:200(IF) |
| Rab anti-c-Myc | Abcam | Cambridge | | Massachusetts | USA | 1:1000（WB） |
| Rab anti-MR | Abcam | Cambridge | | Massachusetts | USA | 1:1000(WB)  1:200(IF) |
| Rab anti-Arg-1 | Abcam | Cambridge | | Massachusetts | USA | 1:1000(WB)  1:200(IF) |
| Mouse anti-CD68 | Invitrogen | Waltham | | Massachusetts | USA | 1:200(IF) |
| Rat anti-F4/80 | Thermo Fisher | Waltham | | Massachusetts | USA | 1:100(IF) |
| Goat anti-β-catenin | biorbyt | San Diego | | California | USA | 1:200(IF) |
| Cy5 anti-mouse-IgG | Invitrogen | Waltham | | Massachusetts | USA | 1:500 |
| Alexa488anti-goat IgG | Invitrogen | Waltham | | Massachusetts | USA | 1:500 |
| Alexa594anti-rab-IgG | Invitrogen | Waltham | | Massachusetts | USA | 1:400 |
| Alexa488anti-rat IgG | Invitrogen | Waltham | | Massachusetts | USA | 1:400 |
| Anti-Ki-67 | Millipore | Bill ricard | | Massachusetts | USA | 1:100 |
